# The roles of RGMa-neogenin signaling in inflammation and angiogenesis

**DOI:** 10.1186/s41232-017-0037-6

**Published:** 2017-03-08

**Authors:** Yuki Fujita, Toshihide Yamashita

**Affiliations:** Department of Molecular Neuroscience, Graduate School of Medicine, Osaka University, 2-2 Yamadaoka, Suita, Osaka, Japan

**Keywords:** RGMa, Neogenin, Immune response, Angiogenesis, Multiple sclerosis

## Abstract

Repulsive guidance molecule (RGM) is a glycosylphosphatidylinositol (GPI)-anchored glycoprotein that has diverse functions in the developing and pathological central nervous system (CNS). The binding of RGM to its receptor neogenin regulates axon guidance, neuronal differentiation, and survival during the development of the CNS. In the pathological state, RGM expression is induced after spinal cord injury, and the inhibition of RGM promotes axon growth and functional recovery. Furthermore, RGM expression is also observed in immune cells, and RGM regulates inflammation and neurodegeneration in autoimmune encephalomyelitis. RGMa induces T cell activation in experimental autoimmune encephalomyelitis (EAE), which is the animal model of multiple sclerosis (MS). RGM is expressed in pathogenic Th17 cells and induces neurodegeneration by binding to neogenin. Angiogenesis is an additional key factor involved in the pathophysiology of EAE. Via neogenin, treatment with RGMa can suppress endothelial tube formation; this finding indicates that RGMa inhibits neovascularization. These observations suggest the feasibility of utilizing the RGMa-neogenin signaling pathway as a therapeutic target to overcome inflammation and neurodegeneration. This review focuses on the molecular mechanisms of inflammation and angiogenesis via RGM-neogenin signaling.

## Background

Repulsive guidance molecule (RGM) is a glycosylphosphatidylinositol (GPI)-anchored glycoprotein with an N-terminal signal peptide, an Arg-Gly-Asp site, a partial von Willebrand type D domain, and a hydrophobic domain of unknown function [1]. RGM was originally identified as an axon repellent in the chick retinotectal system [2, 3]. Neogenin, the receptor for RGM and netrins, is widely expressed in both embryonic and adult tissues and mediates various functions [4, 5]. There are three homologs of RGM in vertebrates: RGMa, RGMb (DRAGON), and RGMc (hemojuvelin). The homologies of chick RGM to mouse RGMa, RGMb, and RGMc are 78, 43, and 40%, respectively.

The binding of RGMa to neogenin regulates axon guidance, neuronal differentiation, and survival during the development of the central nervous system (CNS) [6–8]. Although RGMa expression levels are relatively low in the adult CNS, RGMa expression is induced following ischemic stroke in humans and spinal cord injury in rats [9, 10]. In an animal model of spinal cord injury, treatment with an RGMa-neutralizing antibody at the lesion site significantly enhances axon regeneration and motor function recovery [11]. Because the stimulation of neurons with RGMa induces RhoA and ROCK (Rho-associated coiled-coil-containing protein kinase), resulting in axon growth inhibition, the effect of this antibody may be dependent on the inhibition of this signaling pathway.

In addition to its aforementioned roles, RGMa is involved in neuroinflammatory diseases. The notion that the pathogenesis of multiple sclerosis (MS) is associated with acquired autoimmunity to the CNS has been widely accepted. In MS, immune cells infiltrate the CNS and attack myelin sheaths, leading to demyelination, axonal damage, and neurological disabilities [12, 13]. CD4+ T cells are critical effector cells in CNS inflammation [14]. Interestingly, the inhibition of RGMa via a neutralizing antibody reduces cytokine production, demyelination, and neurodegeneration and relieves neurological deficits in experimental autoimmune encephalomyelitis (EAE) [15, 16]. In addition to its role in neuroimmune interactions, RGMa inhibits angiogenesis, which is often accompanied by inflammation, as mentioned below.

Thus, these findings indicate that the RGM-neogenin signaling pathway is strongly associated with disease severity in neuroinflammatory diseases. In this review, we introduce the pivotal role of RGMa in inflammation and angiogenesis and discuss the potential therapeutic implications of targeting this signaling.

## The RGMa-neogenin pathway mediates autoimmune encephalomyelitis

**Fig. 1:**
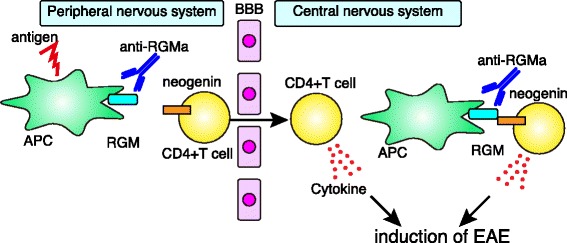
RGMa-neogenin signaling mediates autoimmune encephalomyelitis. RGMa in antigen-presenting cells (APCs) binds to neogenin, leading to the activation of CD4+ T cells in both the peripheral and the central nervous systems. Blocking RGMa with a neutralizing antibody diminishes immune responses and ameliorates the severity of EAE

Although the RGM-neogenin interaction mediates diverse functions in the developing and adult CNS, we also found that RGMa was expressed in bone marrow-derived dendritic cells and that neogenin was expressed in CD4+ T cells. Based on these observations, we assessed the role of RGMa in immune systems and found that the inhibition of RGMa suppressed the T cell response and attenuated the severity of EAE [15]. RGMa treatment of CD4+ T cells induces the activation of the small GTPase Rap1 and increases the adhesion of CD4+ T cells to intracellular adhesion molecule-1 (ICAM-1). Treatment with an RGMa-neutralizing antibody attenuates the clinical severity of myelin oligodendrocyte glycoprotein (MOG)-induced EAE and diminishes relapses in proteolipid protein (PLP)-induced EAE. In humans, an RGMa-specific antibody reduces T cell proliferation and pro-inflammatory cytokine production in peripheral blood mononuclear cells (PBMCs) from individuals with MS. Thus, the RGMa-neogenin signaling pathway is involved in T cell-mediated autoimmune processes in MS (Fig. 1).

**Fig. 2:**
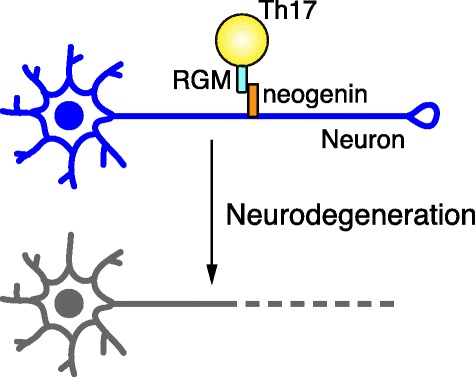
RGMa in Th17 cells induces neurodegeneration. RGMa is preferentially expressed in Th17 cells. The association of RGMa with neogenin in neurons induces neurodegeneration through the dephosphorylation of Akt

Interferon-gamma (IFN-γ)-producing Th1 cells were initially regarded as a predominant effector CD4+ T cell subset that induces the pathogenesis of MS [17]. More recently, interleukin-23 (IL-23) has been shown to be required for the induction of EAE [18] and the pathogenic activity of T helper type 17 (Th17) cells. The key role of IL-17-producing Th17 cells in the pathogenesis of EAE has been established [19]. Indeed, a deficiency of IL-17, IL-17 receptor, or IL-23 receptor diminishes clinical signs in EAE [20–22]. Interestingly, among T cell subsets, including Th0, Th1, Th17, and Treg cells, Th17 cells highly express RGMa. The specific function of RGMa in Th17 cells was determined to be involved in neurodegeneration in EAE [16]. In particular, in Th17 cells, RGMa binds to neogenin and induces Akt dephosphorylation and axonal degeneration (Fig. 2). An RGMa-specific neutralizing antibody diminished neuronal damage and alleviated clinical symptoms of Th17-induced EAE. Taken together, these observations suggest that RGMa could be a therapeutic target for MS. Polymorphisms of RGMa have been correlated with expression changes in IFN-γ and tumor necrosis factor (TNF) in MS patients [23]. This finding raises the intriguing possibility of an association between genetic susceptibility in MS pathogenesis and RGMa.

## Angiogenesis via the RGMa-neogenin pathway

In MS, in addition to various prominent features, such as inflammation, demyelination, and axonal damage, neovascularization is found in inflammatory lesions. In EAE, an angiogenic response is observed following alterations in blood-brain barrier (BBB) permeability and the release of vascular endothelial growth factor (VEGF) [24, 25]. Both detrimental and beneficial effects have been reported in angiogenesis. Since the angiogenic response is related to excess energy consumption and the expansion of inflammation, this response’s pathological contributions to the disease progression of MS and EAE are widely accepted [26]. However, trophic factors from new vessels exert positive effects on the nervous systems. VEGF derived from new blood vessels exhibits pro-inflammatory effects during the early phase of EAE but is involved in repair processes during the late phase of EAE. VEGF mediates the proliferation, migration, and differentiation of neural progenitors and the survival and migration of oligodendrocyte precursor cells [27, 28]. Prostaglandin I_2_ (PGI_2_) produced from new blood vessels is associated with motor recovery in EAE [29]. Thus, specific molecules derived from new vascular cells can be therapeutic targets for MS.

We have shown that RGMa inhibits angiogenesis via neogenin [30]. In the presence of VEGF, RGMa suppresses endothelial tube formation by human umbilical artery endothelial cells (HUAECs), and this effect could be partially reversed by knocking down neogenin. RGMa treatment of HUAECs decreased VEGF-induced phosphorylation of focal adhesion kinase (FAK). It has been demonstrated that netrins, the other ligands of neogenin, also regulate neovascularization. The binding of netrin-4 to neogenin causes neogenin to associate with its co-receptor Unc5b and inhibits angiogenesis both in cultured HUAECs and in an animal model of laser-induced choroidal neovascularization [31]. In contrast, netrin-1 promotes tube formation in HUAECs, and knocking down netrin-1 in zebrafish inhibits vascular sprouting, suggesting that netrin-1 induces angiogenesis [32–34]. However, it is also reported that netrin-1 inhibits angiogenesis via the activation of Unc5b and the disruption of Unc5b induces excess vessel branching and the extension of endothelial filopodia [35, 36]. Netrin-4 binds only to neogenin, whereas netrin-1 is predicted to interact with neogenin, Unc5b, and Unc5c. Differences in binding affinity to neogenin might be responsible for these proteins’ different effects on angiogenesis.

## Conclusions

Here, we reviewed the role of RGMa in inflammation and angiogenesis, particularly in MS. Since RGMa mediates both immune responses and neurodegeneration in EAE, the inhibition of RGMa could be a promising therapeutic intervention for MS. Further research will establish the feasibility of an anti-RGMa antibody for treating MS.

## Abbreviations

BBB: Blood-brain barrier
CNS: Central nervous systems
EAE: Experimental autoimmune encephalomyelitis
FAK: Focal adhesion kinase
GPI: Glycosylphosphatidylinositol
HUAECs: Human umbilical artery endothelial cells
ICAM-1: Intracellular adhesion molecule-1
IFN-γ: Interferon-gamma
IL: Interleukin
MOG: Myelin oligodendrocyte glycoprotein
MS: Multiple sclerosis
PBMCs: Peripheral blood mononuclear cells
PGI_2_: Prostaglandin I_2_
PLP: Proteolipid protein
RGM: Repulsive guidance molecule
Th1: T helper type 1
TNF: Tumor necrosis factor
VEGF: Vascular endothelial growth factor
